# Performance Test of Micro-Slit Antenna Loaded with High Refractive Index Medium Based on Image Recognition

**DOI:** 10.1155/2022/9967681

**Published:** 2022-09-22

**Authors:** Wang Yibo, Yu Bo, Zhang Jinju, Tao Zengjie

**Affiliations:** School of Electronic Science and Engineering, Hunan Institute of Information Technology, Changsha, Hunan 410138, China

## Abstract

Image recognition is the pattern recognition of images. Simply put, it is the prescribed use of the pattern recognition technology in the image. It creates an image recognition template for the input of image information, analyzes and extracts the shape characteristics of the image, and then creates a classifier, relying on the shape of the image classified. However, since particles with high refractive index media can detect galvanic couples and magnetic dipoles together under the excitation of an external field, the interference of the radiation fields of galvanic couples and magnetic dipoles can be used to adjust the incident field. For the study of nanocubes with high refractive index media, the polarization phase of the nanocube particle environment is obtained by the method of long-distance propagation. The loaded micro-slit antenna is also a kind of aperture antenna, which is formed by opening a hole in the metal surface, and the hole will emit electromagnetic waves from the outside of the unit. Loaded micro-slit antennas have a variety of shapes, and they have many advantages such as sturdy structure, fast handling, convenient feeding, and being simple, compact, concealed, and decorative. Therefore, it is necessary to verify various performance parameters of the system by simulating performance tests and to verify the performance of the system by simulating various normal, high load, and abnormal load conditions. The performance test includes load and stress tests, and they can be used together. The purpose of load testing is to clarify the performance of the system under different tasks and to verify the changes in various system performance parameters as the load gradually increases.

## 1. Introduction

The main purpose of image recognition is to resolve and recognize information such as images and texts, so as to solve the direct communication process between the computer and the external environment [1]. The process of image recognition is divided into three stages: text, object, and digital image processing and recognition [2]. In fact, this is the recognition process from simple to structured. It improves the speed of computer performance and improves the corresponding algorithm to make it basic and simple. Image recognition is mainly based on the similarity of “classification” [3]. Under certain conditions, items with the same nature are classified into one category, and objects with different properties are classified into another category. For example, numbers have 10 components, letters have 26 components, and Chinese characters have thousands of components. In addition, different classification methods will lead to different classification results, such as the classification of colors and other characteristics [4]. At present, light field control is still the focus of optical research [5]. With the gradual development of nano-processing technology and nano-material science, more and more attention has been paid to the control of the optical area of micro-nano structures. The reason why the micro-nano structure of high refractive index media can become the focus of research in recent years is because its loss is small, and it also supports magnetic response. In order to determine the regulation effect of the high refractive index medium micro-nano structure on light, we start from the simplest part to study the response of the nano square medium structure to the incident field and the influence of its structural parameters on the control of the light field [6]. In this article, we study the response of the collision site to the isolated nanocube structure, use the internal electromagnetic field mode to carry out the directional scattering of the incident field, and then modify its geometric parameters to obtain the directional scattering. The loaded micro-slit antenna is a kind of antenna that has been gradually developed in the past 30 years. This idea was proposed in 1946, but it was not used in the engineering field. Simple research was carried out from 1950 to 1960, and it was not really developed and used until 1970. The most commonly used type of micro-strip antenna is located on a thin dielectric substrate (such as PTFE laminated fiber), one side is connected to a thin metal layer as a ground plate, and the other side is made by photolithography and etching, using micro-strip lines and axis. The probe feeds the patch, and the loaded micro-slit antenna is thus born. If the patch is a single unit, it is called a micro-strip antenna; if the patch is a thin layer, it is called a micro-strip array antenna; performance testing plays an important role in ensuring program quality. In terms of performance testing, we summarized various solutions and test centers in China to evaluate software performance from three aspects: client application testing, network creation, and server creation testing. Various solutions and test centers evaluate the performance of the software in three aspects: client application testing, network creation, and server creation testing. Under normal circumstances, through the combination of three aspects, the system performance can be comprehensively analyzed and predicted.

## 2. Related Works

The literature introduces the scattering properties of nano-dielectrics and proves the resonance of electric and magnetic dipoles [7]. By selecting the appropriate wavelength to form an electric dipole shape and a magnetic dipole shape of the same size and shape, scattering is achieved. By changing the geometric dimensions of the object, the electric and magnetic fields are combined to achieve unidirectional scattering at the resonance position [8]. In addition, by linking the above-mentioned particles into chains, a broad spectrum of unidirectional scattering can be performed. The literature introduces the interaction between nanodimer dielectric and Gaussian beam and proposes a nanometer displacement measurement method. By measuring the scattered field, the position of the nanodimer corresponding to the center of the collision area can be obtained [9]. The measurement accuracy of this method can reach 10 nm, and HG10 can be used to improve the measurement accuracy. The literature introduces the consistent combination of metasurfaces, the organization of metasurfaces in a systematic way, and the association of its unique Jones matrix [10]. Unlike optically active materials that increase the rotation angle by increasing the length of the propagation direction, the metasurface can achieve the rotation angle by changing the size of the rear part of the structure, and the deviation angle ranges from -90° to +90° [11]. The literature introduces a limited range of Boltzmann machines and SVM to build a multi-layer classification model, uses deep learning methods to extract pattern samples, and then uses SVM methods to classify and apply them to visual recognition work [12]. Experiments with fewer samples confirm that the comparison between vector machines and deep trust networks is better, and after comparing the number of samples, layers, nodes, and accuracy, the relationship between the number of nodes in the hidden layer and the number of support vectors is discussed [13]. The literature introduces the research background and significance of dual-frequency micro-strip antennas. It also explains in detail the electrical parameters and basic theories of micro-strip antennas, the basic theories of slit antennas, and the basic theories and features of dual-band antenna design. Next, this article improves the structure of the dual-frequency micro-strip antenna and analyzes its various structural parameters in detail in the HFSS simulation program [14].

The literature describes deep learning systems. It is an advanced machine algorithm that is based on learning to represent complex relationships between simulated data. Observation results can be expressed in many ways, such as using a series of rated powers to represent pixels. Unique representation methods can speed up the algorithm to complete learning activities (such as facial recognition). The purpose of characterization learning is to find a better representation method. By simulating the brain structure and nervous system similar to the human brain, and the data are gradually being developed to form a more visible representation (features or parts). Deep learning uses different layers of indirect information processing to enable or manage image capture and conversion, format analysis and classification, and to interpret data such as images, sounds, and text. High-level forms and concepts are defined by sub-concepts and sub-characteristics. The same sub-concept can be used to identify multiple high-level concepts. Such a structure is also called a deep-level structure [15]. The literature introduces that the convolutional neural network is composed of related components and combined lines of a complete integrated line. With this system, CNN can use a two-dimensional data input system, but compared with other deep regions, the custom network shows better results in audio applications [16]. The conversion network can also be trained using standard replication algorithms. Due to its small scale, it can be easily trained from other deep models.

## 3. Design of Performance Test Model for Micro-Slit Antenna Loaded with High Refractive Index Medium Based on Image Recognition

### 3.1. Image Recognition Technology

Boltzmann's deep machine is a binary pairwise undirected probability graph model (i.e., Markov random field) and has a wide range of anonymous segments and a network of interconnected units. In RBM, there is no connection between hidden layers and between visible layers. The criteria assigned to the visible layer are

(1) $$\begin{array}{c} p\left(v\right)=\frac{1}{Z}\underset{h}{\sum }e^{\underset{ij}{\sum }W_{ij}^{\left(1\right)}v_{i}h_{j}^{\left(1\right)}+\underset{jl}{\sum }W_{ij}^{\left(2\right)}h_{j}^{\left(1\right)}h_{l}^{\left(2\right)}+\underset{m}{\sum }W_{m}^{\left(3\right)}h_{l}^{\left(2\right)}h_{m}^{\left(3\right)}}\text{.} \end{array}$$

Like DBN, a deep Boltzmann machine can use a limited amount of labeled data to improve the characterization of a large amount of unlabeled data, so that it can check complex performance and summary. Different from deep convolutional neural network and DBN, DBM uses a two-way training method and reasoning, that is, transfer from top to bottom and from bottom to top, so that the system can better display the fuzzy and complicated feature. In DBN, the uppermost RBM is an undirected graph model, and the bottom layer consists of a directed model. As a DBM, it is difficult to obtain the maximum possible probability. We can use the most reasonable estimate or use the mean field inference model to estimate the probability of the data and use it with the Markov chain Monte Carlo model according to the random estimation method. However, because the estimation reasoning is based on the standard site method, it is 25–50 times slower than the following DBN model, which makes its application unsuitable for many types.

In a single RBM, assuming that the pixel corresponds to the visible layer as *v*, there is *n* nodes; the obtained shape corresponds to the hidden layer as *h*, there is *m* nodes; the visible layer and the hidden layer system (*v*, *h*) have energy as follows:

(2) $$\begin{array}{c} E\left(v,h\right)=-\overset{n}{\underset{i=1}{\sum }}a_{i}v_{i}-\overset{m}{\underset{j=1}{\sum }}b_{j}v_{j}-\overset{n}{\underset{i=1}{\sum }}\overset{m}{\underset{j=1}{\sum }}v_{i}w_{ij}h_{j}\text{.} \end{array}$$

It can be seen that by expanding and normalizing the energy performance, the joint probability distribution of the unit vector of the visible layer and the hidden layer in the vector can be obtained:

(3) $$\begin{array}{c} p\left(v,h\right)=\frac{1}{Z}e^{-E\left(v,h\right)}\text{.} \end{array}$$

In the formula, *Z* represents the normalization constant, adding all pairs of visible and hidden layers:

(4) $$\begin{array}{c} Z=\underset{v,h}{\sum }e^{-E\left(v,h\right)}\text{.} \end{array}$$

The possibility for the network to provide visible layer vectors is to include all visible layer vectors in the layer:

(5) $$\begin{array}{c} p\left(v\right)=\frac{1}{Z}\underset{h}{\sum }e^{-E\left(v,h\right)}\text{.} \end{array}$$

Because the visible layer vector and the hidden layer vector are independent of each other, there are

(6) $$\begin{array}{c} p\left(h\;|\;v\right)=\overset{m}{\underset{j}{\prod }}p\left(h_{j}\;|\;v\right)\text{,}\\ p\left(v\;|\;h\right)=\overset{n}{\underset{i}{\prod }}p\left(v_{i}\;|\;h\right)\text{.} \end{array}$$

If the visible layer *v* is given, the probability that the binary state of *v* is 1 is

(7) $$\begin{array}{c} p\left(h_{j}=1Iv\right)=\sigma \left(b_{j}+\overset{n}{\underset{i=1}{\sum }}v_{i}w_{ij}\right)\text{.} \end{array}$$

If the hidden layer *h* is given, the probability that the binary state of *v* is 1 is

(8) $$\begin{array}{c} p\left(v_{i}=1\;|\;h\right)=\sigma \left(a_{i}+\overset{m}{\underset{j=1}{\sum }}w_{ij}h_{j}\right)\text{.} \end{array}$$

In the reverse probability vector obtained through this step, the result is used as the weight increment between the input layer and the output layer. In each case, the weight increment and the bias update are the same at about 1*w*.

By sorting out and summarizing the algorithms above, different training samples are selected for comparative testing, and the results are shown in Tables 1–3.

Table 1 tests the test accuracy of a single hidden layer, when the number of samples is 1000/200, and when the number of samples is 5000/1000, the test results are shown in Table 2.

Table 3 summarizes the research results in Tables 1 and 2 and analyzes the corresponding prediction accuracy when the design has two hidden layers.

**Figure 1:**
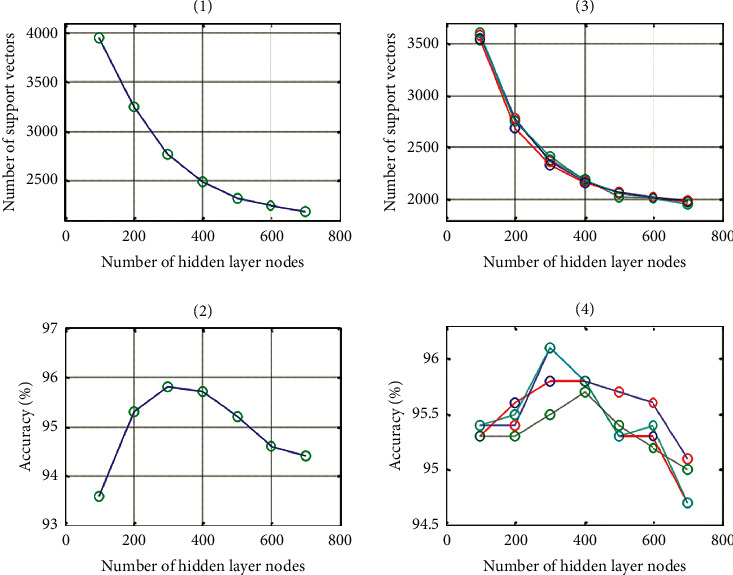
The relationship between the number of hidden layer nodes and the number of support vectors and the accuracy of prediction.

By comprehensive analysis of Tables 1–3, it can be seen that the increase in the number of samples can promote the improvement of accuracy, and the prediction accuracy of multiple hidden layers is higher than that of a single hidden layer. The relationships are shown in Figure 1.

This test has a different *C* value. In this experiment, the number of training samples is 5000, the number of test samples is 1000, other parameters are unchanged, and the *y* value is a standard instrument of 0.0033. Take different *C* values and compare the accuracy of training samples, the number of support directions, and the accuracy of test samples of the *C*-to-SVM method and the RBM-SVM method, as shown in Table 4.

The RBM-SVM method is used for a single hidden layer, and the number of hidden layer nodes is 300. The experimental results are shown in Table 5.

**Figure 2:**
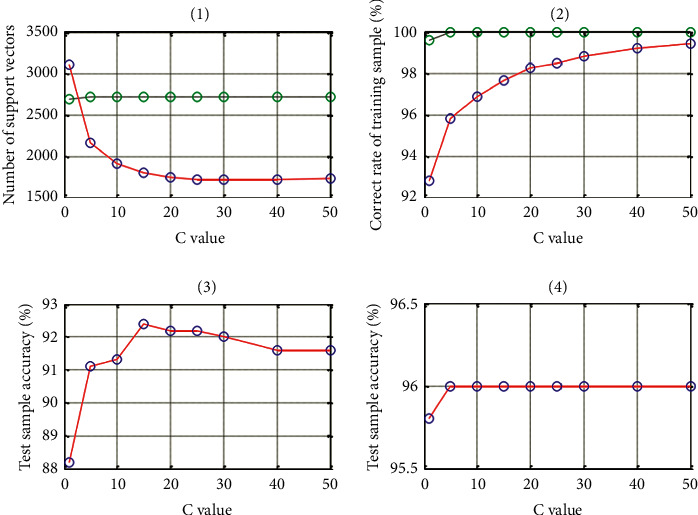
The effect of penalty factor *C* value on accuracy.

As shown in Figure 2, the straight line uses the SVM method, and the dotted line uses the RBM-SVM method. For *C*, the SVM method and RBM-SVM method correspond to the number of support. As shown in the figure, the value of *C* increases. For the RBM-SVM method, the number of support vectors increases slightly, the number of parasites decreases significantly, and then becomes flat. There is almost no change in development. For the SVM method, the number of support vectors accelerates and decreases and then gradually develops with slight changes.

### 3.2. Mode Analysis of High Refractive Index Medium

Now suppose that the scattering part of the measurement length is placed in an infinite environment of space and is directly illuminated with a combined wavelength.

(9) $$\begin{array}{c} E_{s}=E_{MD}+E_{ED}+E_{MQ}+E_{EQ}+\ldots \text{.} \end{array}$$

*Quadrupole Power (EQ)*. If the particle size is smaller than the length, the radiation length of the long-order end is much smaller than the radiation length of the dipole and quadrupole, which can be ignored. Sometimes, we can ignore the four-level mode and consider the dipole mode, which is called dipole approximation. The corresponding vector of the polar radiation plant is expressed as

(10) $$\begin{array}{c} E_{MD}=-\eta_{0}\frac{k^{2}}{4\pi }\frac{e^{ikr}}{r}n_{r}\times m\text{,}E_{ED}=\eta_{0}\frac{ck^{2}}{4\pi }\frac{e^{ikr}}{r}n_{r}\times p\times n_{r}\text{,} \end{array}$$

(11) $$\begin{array}{c} E_{MQ}=\eta_{0}\frac{ik^{3}}{24\pi }\frac{e^{ikr}}{r}n_{r}\times Q_{MQ}\text{,}E_{EQ}=-\eta_{0}\frac{ick^{3}}{24\pi }\frac{e^{ikr}}{r}n_{r}\times Q_{MQ}\times n_{r}\text{.} \end{array}$$

It can be seen from formulas (9–11) that in order to find the scattering area of the incident field, it is necessary to know the magnitude of the internally induced polar moment. The magnitude of the polar moment is determined by its own polar quantization, the Eoc of the local power plant, and the regional magnetic field. Take the dipole moment as an example:

(12) $$\begin{array}{c} p={\bar{\bar{\alpha }}}_{ee}E_{loc}+{\bar{\bar{\alpha }}}_{em}H_{loc},m={\bar{\bar{\alpha }}}_{me}E_{loc}+{\bar{\bar{\alpha }}}_{mm}H_{loc}\text{.} \end{array}$$

**Figure 3:**
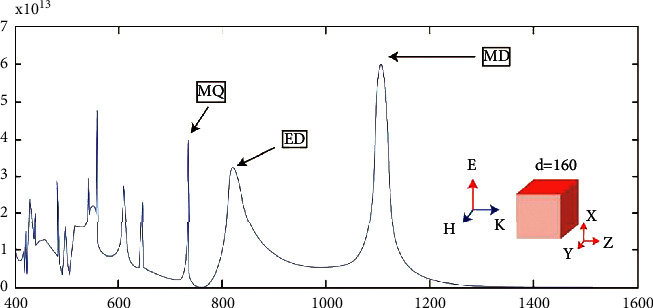
Numerical simulation results of scattering spectra of nanocubes.

As shown in Figure 3, the magnetic field distribution in the plane xy refers to the upper and lower films, and the orientation is opposite, forming a circular distribution in the plane yz, so the electric field along the *x* direction is induced, corresponding to the distribution in A circular spot in the cy plane. The distribution of this form of electromagnetic field is a form of electric dipole radiation polarized along the *x* direction, which represents the electrode peak corresponding to the corresponding resonant peak.

### 3.3. Micro-Slit Antenna

As a new type of antenna, micro-strip antennas have developed slowly in the past 30 years. This idea was proposed as early as the beginning of 1946, but it did not attract the attention of the engineering field. There was only a small amount of research from 1950 to 1960, and the real development and use was in 1970. The common type of micro-strip antenna is suspended on a thin medium (such as Platinla laminated fiber), one side is connected to a thin metal layer as a ground plate, and the other side is made of a specific metal frame. Use the micro-strip line and the axis probe to feed, and this is the composition of the micro-strip antenna. Compared with ordinary micro-strip antennas, micro-strip antennas have many advantages:

1. Small size, light weight, low profile, and conformal to the carrier.
2. The electrical properties are diverse, and different components are easily available.
3. Easy to install. It can be used in combination with active devices and integrated circuit boards.

Due to the inherent advantages of micro-strip antennas, it is now widely used in many high-precision fields such as satellite phone communications, aircraft antennas, Doppler radar, and missile telemetry.

The slit antenna is an antenna aperture antenna that is generated by the open position of the metal surface and the electromagnetic wave emitted from the external position. In 1953, H. G. Booker proposed the concept of slit antenna. Slit antennas have a variety of shapes and have many advantages: solid structure, fast processing, convenient feeding, compactness, low profile, good hiding and good decoration, and many other advantages, making it an important communication field including 3G technology, LAN, wireless network, and other important applications.

**Figure 4:**
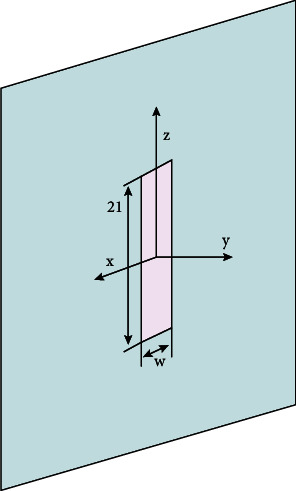
Structure diagram of slot antenna.

Figure 4 shows a diagram of the overall structure of a general slit antenna. In an ideal state, a narrow slit can be opened, which can be regarded as two different dipole antennas; assuming the length is 2*L* and the width is *w*, (2*L* >> *w*).

The electric field is perpendicular to the gap, which can be expressed as

(13) $$\begin{array}{c} E_{x}=\frac{U_{ms}}{w}\sin\;k_{0}\left(l-\left|z\right|\right)\text{.} \end{array}$$

In the formula, *U*_*ms*_ is the antinode voltage. At the corresponding height of the electromagnetic zone and the electromagnetic wave, the radiation can be expressed in the same magnetic direction in the *z*-axis direction where the surface of the gap is located, and the surface density of the magnetic field can be displayed, as shown in the following formula:

(14) $$\begin{array}{c} J_{ms}={\hat{z}}^{2}\frac{U_{ms}}{w}\sin\;k_{0}\left(l-\left|z\right|\right)\text{.} \end{array}$$

Assuming that the slit magnetic current is uniformly distributed on *x*, the slit magnetic current can also be expressed as

(15) $$\begin{array}{c} I_{ms}=2U_{ms}\sin\;k_{0}\left(l-\left|z\right|\right)\text{.} \end{array}$$

If the principle of equivalence is used, the correct position can only be calculated on one side of the space and zero on the other side. If the magnetic current is used as the hypothetical source, if the gap area is found on one side, the field on the other side of the field is found symmetrically. The local radiation can be obtained directly from the dipole antenna using the principle of the double-sided electromagnetic zone, and the results are as follows:

(16) $$\begin{array}{c} \begin{array}{c} {\overset{⟶}{A}}_{m}\left(r\right)\approx \frac{\varepsilon_{0}}{4\pi r}e^{-jk_{0}r}\int {\overset{⟶}{J}}_{m}\left(z^{\prime }\right)e^{jk_{0}r^{\prime }}dv^{\prime }=\frac{\varepsilon_{0}U_{ms}\overset{⟶}{z}}{\pi k_{0}r}e^{-jk_{0}r}\frac{\cos\;\left(k_{0}l\;\cos\;\theta \right)-\cos k_{0}l}{\sin^{2}\;\theta }\text{,}\\ H_{\theta }=-j\omega A_{ms}=j\frac{U_{ms}}{\eta_{0}\pi r}\frac{\cos\;\left(k_{0}l\;\cos\;\theta \right)-\cos k_{0}l}{\sin\;\theta }e^{-jk_{0}r}\text{,}\\ E_{\varphi }=-H_{\theta }\eta_{0}=-j\frac{U_{ms}}{\pi r}\frac{\cos\;\left(k_{0}l\;\cos\;\theta \right)-\cos k_{0}l}{\sin\;\theta }e^{-jk_{0}r}\text{.} \end{array} \end{array}$$

According to Maxwell's equation and given conditions, the electric field of the symmetrical dipole antenna can be obtained:

(17) $$\begin{array}{c} E_{\theta t}=j\frac{I_{m\;d}}{2\pi r}\sqrt{\frac{\mu }{\varepsilon }}e^{-jkr}f\left(\theta \right). \end{array}$$

For a half-wave ideal slit antenna, 2*l*=*λ*/2, *kl*=*π*/2; therefore,

(18) $$\begin{array}{c} f\left(\theta \right)=\frac{\cos\;\left(\pi /2\cos\;\theta \right)}{\sin\;\theta }\text{.} \end{array}$$

Both have the same radiation field, and the radiation power of the symmetrical dipole antenna is

(19) $$\begin{array}{c} P_{rd}=\frac{1}{2}I_{m\;d}^{2}R_{rd}\text{.} \end{array}$$

The radiation power of an ideal slit antenna is

(20) $$\begin{array}{c} P_{rs}=\frac{1}{2}U_{ms}^{2}G_{rs}\text{.} \end{array}$$

Therefore, the input conductance of an ideal slit antenna can be expressed as

(21) $$\begin{array}{c} G_{as}=\frac{G_{rs}}{\sin^{2}\;\left(kl\right)}\text{.} \end{array}$$

The results obtained by the above calculation method show that the antenna shape of the finite plane has the following characteristics: the slit of the slit antenna has no radiation on the slit axis, and the size of the structured plane affects the radiation amount and position of the antenna. The shape of the emission plane has a negative influence on the antenna position in this direction, so the exact position of the plane (i.e., the shape of the H plane) is no different from an ideal antenna.

## 4. Performance Test Practice of Micro-Slit Antenna Loaded with High Refractive Index Medium Based on Image Recognition

### 4.1. Antenna Structure

The geometric structure of the modified antenna is different from the original antenna in that a structural branch structure is added at the end of the feeding micro-strip, and a metal bottom is added at the bottom. Adding branches to the sector terminal makes it easier and more adjustable to connect the antenna. The metal material improves the efficiency of the original antenna and improves the radiation performance.

The upper part of the dielectric substrate is a ground plane, a radiation gap is drilled on the ground plane, and on the other side is a micro-strip line with multiple terminal branches for power supply. A 40 mm × 40 mm metal substrate was added to the place below 8 mm from the dielectric device to improve the performance of capture and radio antennas.

### 4.2. Antenna Improvement Analysis

For the antenna area, micro-strip line feed and coaxial feed are used. The antenna in this article is a micro-strip line feeding method. At the end of the micro-strip line, the terminal branch structure of this structure is used to improve antenna reception. The continuous and effective dielectric is a, the width of the micro-strip line is *w*, the thickness of the dielectric substrate is *h*, *s* represents the frequency of the electric field, and *u* and *s* represent the permeability and permittivity of the electric field, respectively. Therefore, in a micro-strip line with a dielectric thickness and a constant dielectric, the width becomes the only condition for its characteristic impedance. Because of the existence of the terminal branch, it changes the original part of the antenna radiation. Adjusting the angle and length of the terminal branch can make the overall radiation of the antenna close to zero, while ensuring that the internal power of the antenna is free from oscillation. The use of radiation solves the problem of imbalance between the antenna and the feeding point. In the following, simulation analysis will be carried out on the influence of fine-tuning of the impedance of the fan-shaped terminal stub.

The antenna gain in the article is −10 dB and -2 dB at 2.4 GHz and 5.2 GHz, respectively. This shows that most of the energy transmitted by the wire transmission to the antenna is not well radiated in the form of electromagnetic waves but is consumed in the antenna. In order to solve this problem, the component first uses (0.002) F4B material with very low dielectric loss and then adds a metal substrate larger than the antenna size to the lower part of the antenna to achieve the mirror image effect and make it have good radiation performance.

The use of low dielectric constant devices can effectively reduce the loss of electromagnetic waves in the medium transmission process and improve the radiation efficiency of the antenna. The role of the metal bottom is to change electromagnetic radiation from downward radiation to upward radiation.

### 4.3. Simulation Analysis

The previous article introduced four kinds of electromagnetic simulation software. This topic uses Ansoft's HFSS electromagnetic simulation tool as the main simulation software. In HFSS, modeling is carried out according to the design structure scheme, and it is used when waiting for the next optimization. It is impossible to design an antenna once, and every change of antenna parameters must undergo several tests to gradually approach the most effective result. In most cases, several parameters need to be tuned together to produce close to ideal results. The simulation results of multiple parameters will be given below. These results have the greatest impact on the results of the antenna simulation process.

**Figure 5:**
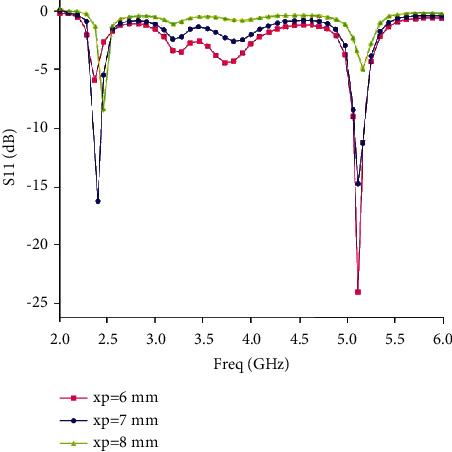
Influence curve of micro-strip line position on S11.

This antenna can successfully perform dual operations because the micro-strip feeder is at a different distance from the two main radiation fields. Since different positions are found in the feed, these two positions adapt electromagnetic waves to different waves.  Figure 5 shows the simulation result of xp = 6, xp = 7, and xp = 8 under the condition of observing other boundaries.

From the figure, xp plays an important role in the doubling process. A little change will cause a sudden change in one of the frequency points and lose its dual-frequency characteristics. Taking xp = 7 mm as the midpoint, if xp becomes larger, the upper wave and the lower wave will also move with the change of the upper wave. Two or more changes will not only cause the position of the repeated points to change but also make the combination of two repeated points worse. If xp = 7 mm, you can see the ideal situation.

**Figure 6:**
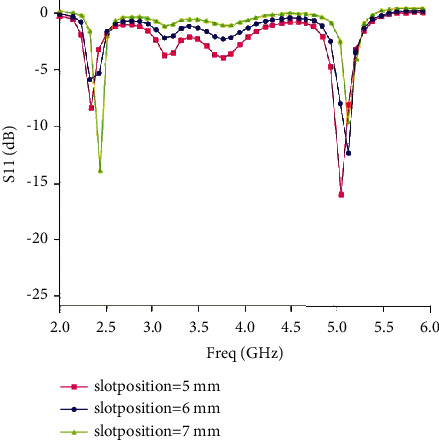
Influence curve of gap position.

In the latest analysis, micro-strip line feeding is a one-time feeding method. At the same time, the distance between the feeding micro-strip and the radiating unit must be correct, and repeated attention and caution must be exercised. In slit antennas, the main effect of radiation is the slit, which will naturally have a significant impact on the antenna performance.  Figure 6 is a graph showing the result of the difference in *x*-axis length.

From Figure 6, changing the position of the *y*-axis will also change the performance of the antenna. However, unlike xp at the xp distance in the micro-strip feeder, the change of sp usually affects the position of the frequency resonance. If sp decreases, the matching degree of the low frequency becomes worse, and the matching degree of the high frequency becomes better; this can be clearly seen in the above figure (sp = 5 mm and sp = 6 mm). Considering the relevance of repetition and consistency, sp = 6 mm is selected as the standard setting for the following simulations.

### 4.4. Optimization Results

For each parameter, the method of obtaining the result is adopted, that is, whenever the other parameters are unchanged, a unit will be changed to a specific value. Each value represents a result. If the result is close to the optimal condition, the corresponding result is regarded as the ideal value. According to the above method, each parameter that may lead to the change of the composite distance is studied separately, and finally the best combination of parameters is obtained, as shown in Table 6.

## 5. Conclusion

In summary, by studying the origin of high refractive index media, this article introduces a new type of optical fiber sensor, which has a very good prospect. The first part of this article introduces the research background and importance of high refractive index media and further understands the application aspects of high refractive index media. Then, we summarized and studied the status quo and progress of liquid level sensing and temperature sensing and understood their progress and differences. It lays a solid foundation for the subsequent high refractive index medium in liquid level and temperature sensing. Next, we studied the basic viewpoints of long-term optical fiber networks and analyzed several characteristics of optical fibers. First, the integrated structure theory of its long-period optical fiber network has been systematically analyzed to fully understand the principle of long-period fiber packaging. Second, in-depth study of the directional patterns, flow patterns, and radiation patterns in the optical fiber will lay a solid foundation for the next simulation analysis; then, this article describes the origin and importance of the micro-strip antenna research and briefly summarizes what the outside world uses (four kinds of simulation software). Third, this article summarizes the electrical parameters of antennas, the basic views of slit antennas and micro-strip antennas, and introduces many methods commonly used to achieve dual frequency. Finally, this article improves the double U-slit antenna and conducts a detailed simulation analysis. This article summarizes the antenna parameters used in antenna production and the structural influence of each component, such as S11 and gain. It also summarizes the steps of visual recognition, highlights common methods and current problems related to image acquisition and image recognition, and deepens the status quo and concepts of deep learning, as well as the benefits of development and research. Also, we carried out the study of Boltzmann's principle and its limitations and the study of Boltzmann machine conversion, expounding many common methods and the basic structure of deep intelligence. Convolution neural network is the research result of neural system. The stimulus signals are transmitted through the nervous system of the brain and rely on their own functions, such as low connectivity, reduced sampling, and so on, and have shown self-learning in various cognitive functions. In particular, if the input is a two-dimensional image, a consistent network structure can widely spread the original information part of the visualization layer (invisible layer) and capture the parallel movement and rotation of a specific layer. We found that the optical architecture is compatible, so the antenna can work in the two frequency bands 2.4 GHz and 5.2 GHz. Finally, based on the simulation analysis, this antenna was designed, and after testing, the simulation combination for the expected purpose was realized.

## Figures and Tables

**Table 1:** The prediction accuracy of different methods under a single hidden layer (%) (number of samples: 1000/200).

Method	Number of hidden layer nodes						
	100.00	200.00	300.00	400.00	500.00	600.00	700.00
DBN	91.5	92.5	94	94.5	92.5	93	93
RBM-SVM	92	93.5	93.5	93	93	93	93
SVM	82	82	82	82	82	82	82

**Table 2:** The prediction accuracy of different methods under a single hidden layer (%) (number of samples: 5000/1000).

Method	Number of hidden layer nodes						
	100.00	200.00	300.00	400.00	500.00	600.00	700.00
DBN	93.5	92.5	93	93	92.5	92	97
RBM-SVM	96	97.5	92.4	90	91	96	91
SVM	88.1	88.1	88.1	88.1	88.1	88.1	88.1

**Table 3:** The prediction accuracy of each method when there are two hidden layers (%).

Method	Number of hidden layer nodes						
	100.00	200.00	300.00	400.00	500.00	600.00	700.00
DBN	92.4	91.5	92	92	91.5	91	96
RBM-SVM	95	96.5	91.4	91.2	91.4	96	91.7

**Table 4:** Relationship between C value and study accuracy when using support vector machine approach.

*C* value	1	5	10	15	20	25	30	40	50
Number of support vectors	3110	2155	1908	1806	1740	1712	1712	1715	1725
Test sample prediction accuracy	88.1	91.2	91.4	92.3	92.3	92.3	91	91.5	91.5
Training sample prediction accuracy	92.7	95.76	96.85	97.61	98.25	98.4	98.83	99.21	99.5

**Table 5:** The influence of method C value on accuracy in this paper.

*C* value	1	5	10	15	20	25	30	40	50
Number of support vectors	2681	27166	2718	2718	2718	2718	2718	2718	2718
Test sample prediction accuracy	95.9	97	97	97	97	97	97	97	97
Training sample prediction accuracy	99.57	100.00	100.00	100.00	100.00	100.00	100.00	100.00	100.00

**Table 6:** Optimized parameters.

Parameter	Numerical value	Remarks
ws	35 mm	Media board width (*x* direction)
Is	24 mm	Media plate length (*y* direction)
xp	7 mm	Distance from bottom micro-strip line to origin
istrip	8.3 mm	Micro-strip line length (*y* direction)
wstrip	1.7 mm	Micro-strip line width (*X* direction)
wl	12.5 mm	Two U-shaped gap distances
slitposition	6 mm	The distance between the bottom of the gap and the origin
ss	1.5 mm	Gap width in *Y*-axis direction
sh	2 mm	*X*-axis direction gap width
Theta	70°C	The angle between one side of the fan-shaped terminal branch at the front end of the micro-strip line and the *X*-axis
w2	3.5 mm	The distance between two U-shaped gaps
Istub	2 mm	The length of the fan-shaped terminal stub at the front end of the micro-strip line
hg	8 mm	The distance between the bottom metal plate and the dielectric plate (*Z* direction)
wg	66 mm	The width of the bottom metal plate (*X* direction)

## Data Availability

The data used to support the findings of this study are available from the corresponding author upon request.
